# Clinical Review, and Hospital Reports

**Published:** 1838-10-01

**Authors:** 


### Clinical Review.

### GUY'S HOSPITAL.

#### I. Experiments and Observations on Albuminous Fluids. By Dr. Babington.

The following observations of Dr. Babington's appear to us to be highly valuable. They are intended to illustrate the changes which albuminous fluids undergo by an union with the pure alkalies and the neutral salts.

Urine which contains albumen, or pus, is, for the most part, acid. But urine which contains adhesive, ropy mucus, is almost invariably alkaline. The latter is formed by the mucous membrane of the bladder in the state of chronic inflammation. But the researches of Dr. Babington, which we shall now quote, go to prove the existence of a closer affinity between these two deposits than was previously imagined.

"During an attendance," says Dr. Babington, "some months since, on a gentleman who had calculus in the bladder, together with prostatic disease, I had frequent occasion to examine the condition of his urine; which, under the use of acidulated infusion of Buchu, maintained an acid re-action, and was in itself clear and of a natural colour. Towards the end of micturition, however, more especially when first performed in the morning, a cream-coloured opake fluid was voided; which became mixed with the urine, and ultimately settled at the bottom of the vessel in which it was received. This fluid was not ropy; so that on decantation, it could be made to flow off, drop by drop; and when thus divided, it appeared to consist of minute flocculi. The application of heat caused an immediate flocculent precipitate; and alcohol, acids, and all such agents as precipitate albumen, had the same effect. On the addition of a moderately strong solution of pure potassa, a remarkable change occurred: the thick creamy sediment became transparent; and was converted, on agitation, into a semi-solid mass, so viscid and tenacious, that, in pouring it from one test-tube to another, a continuous string, several feet in length, could be formed. This mass I found to be of difficult solubility in water; the mere affusion of that menstruum being wholly insufficient, even after many days maceration, to alter its character, or abstract the alkali from it. It was, therefore, practicable to wash its surface, so as to free it from any alkaline re-action upon turmeric or reddened litmus-paper. It bore a boiling heat, without losing its viscid consistence or transparency; and remained unaltered, in an open phial, for several weeks. Nitric acid, much diluted, only rendered its surface opalescent; but when added in a more concentrated form caused a precipitate. Ammonia was equally efficacious as the fixed alkalies, in forming with the urinary sediment a tenacious transparent semi-fluid. This strongly retained the ammoniacal odour; and the compound bore a similarity so striking to natural ropy mucus passed under certain diseased states of the bladder, that I was induced to institute a more minute comparison between them. The opportunity of doing so was afforded me by a case of diseased bladder, with catarrh of that organ, under my care at the same period. The urine in this instance was of a deep colour, and not very clear: it had an ammoniacal odour, was highly alkaline, and deposited a viscid transparent amber-coloured mucus. This mucus was as ammoniacal in its re-action as the urine itself. It was difficultly soluble in water. The caustic alkalies rendered its odour more pungent, but did not affect its viscidity or general appearance. Nitric acid caused a copious precipitation of brown flocculi, insoluble in water. From these characters, this natural ropy mucus seemed to me closely to resemble, if not to be identical with, the artificial combination with ammonia which I had effected, in the first case, by the union of ammonia and the urinary sediment. That sediment I considered to be of a purulent nature; but coming, as it did, from a hidden source, direct proof of this was wanting; and my next endeavour, therefore, was to ascertain whether the same combination could be formed by a union of the pure alkalies with matter from other parts. Fresh pus, from an abscess in the groin, was made the subject of experiment. It was slightly acid; and on the addition of liquor potassæ, just sufficient for saturation, it began to thicken, and become viscid. A further addition of alkali, brought in contact with it by agitation, effected its complete conversion into a semi-fluid transparent tenacious mass. Solutions of soda and of ammonia produced a similar change; the latter, indeed, in so remarkable a degree, that whilst the pus, when poured into a clean test-tube, was, in accordance with its usual characters, a thick opake creamy fluid, adhering, as it flowed, to the sides of the glass, on being agitated with liquor ammoniæ it immediately formed a tough transparent semi-solid, which could with difficulty be shaken towards the mouth of the tube at all; but on being caught and contorted as it protruded, the whole was completely brought away, even from the very bottom, so as to leave the internal surface of the tube as clean and free from moisture as before the pus was poured into it. The mucous expectoration in pneumonia will, in the same way, leave the surface of the vessel which contains it. The combination thus formed was very difficultly soluble in water, and retained its viscidity and alkaline qualities in its internal substance, although repeatedly washed: it also preserved its ropiness and transparency, even though surrounded by a weak acid solution more than sufficient to neutralize the alkali with which it was combined. A strong solution of nitric acid rendered the mass opake, and destroyed its elasticity. Upon the whole, this combination was of the same nature, though more concentrated than that which I had first obtained by the combination of alkalies with the purulent urinary sediment.

As it is often a matter of doubt with the medical man, whether a deposit in urine be of a purulent nature or not, it is a ready test, after pouring off the clear supernatant urine, to add liquor potassæ to the sediment collected in a phial or test-tube. If it be purulent, it will, on agitation, form, with the alkali, the transparent viscid compound which I have described. This I have more than once verified, since my attention was first drawn to the subject."

After detailing a variety of experiments, and building on them several ingenious observations, Dr. Babington terminates the paper with the following conclusions:—

That, probably, natural mucus is formed by some combination analogous to that which results from the action of a pure alkali, or of a neutral salt, on pus or albumen, either by actual chemical combination, or by catalytic force: in which latter case, the proportion of the alkali or salt may be of the less consequence.

The intimacy of the union in the artificial combination is so great that it is not destroyed by an acid, provided this be largely diluted, even though it be added in quantity quite sufficient to saturate the alkali, were it in an uncombined state. This holds true of natural mucus, when secreted in the bladder; for Sir B. Brodie has remarked, that the ropy mucus, secreted under a morbid condition of that viscus is alkaline, while the urine secreted at the same time occasionally remains acid.

The utility, he goes on to remark, of this power of resisting decomposition is manifest in a fluid which is destined to defend surfaces from the contact of injurious agents. For the purpose of lubrication, too, it is difficult to conceive any combination more smooth and slippery than are those containing a free alkali. Liniments and soaps are the compounds to which we have recourse, when we wish to obtain these qualities in the highest degree.

He adds, what indeed appears singular :—

"In the albuminous urine of renal dropsy I have endeavoured in vain to obtain, by the addition of alkalies, any approach to ropy mucus ; but whether this is owing to some counteracting tendency in the other constituents of the urine, or to some alteration in the albumen itself, or whether the proportion is too small to render its viscidity palpable, I have not ascertained. To obviate the last cause of failure, I have concentrated such urine, by evaporation, at a temperature too low to produce coagulation ; yet still I had no better success."

These observations of Dr. Babington are confirmed and carried out by a most promising young chemist, Mr. Golding Bird. This gentleman bids fair to take a high position in the walks of animal chemistry. He has published a long, an elaborate, and a very able paper on the "Chemical Nature of Mucous and Purulent Secretions," in the sixth number of the Guy's Hospital Reports. From that paper we can do no more, and we should do no less, than extract the concluding observations, which bear upon the questions mooted in the paper of Dr. Babington.

"If the facts advanced in the two preceding sections are proved, by subsequent observers, to be universally correct, we cannot but admit their importance, even if they only serve to point out the analogy between mucous and serous surfaces, as evinced in their secretions. And, if, as has been shewn, *albumen* can readily, under certain circumstances, become converted into mucus, so we no longer have any difficulty in understanding how mucous membranous surfaces may, under certain states of irritation, pour out albumen in a free or coagulated state. Thus, if the lining membrane of the larynx and trachea—which presents, normally, a surface secreting genuine mucus—be considered as pouring out the albuminous particles of the blood combined with an excess of saline matter (thus constituting mucus ?), we have no difficulty in understanding how the same membrane may, from incidental circumstances, pour out the albuminous particles of blood, combined with but a small proportion of saline matter, constituting that form of secretion to which the term 'lymph' is applied—a secretion capable of taking on organization, in which particular it physiologically and essentially differs from mucus. If this hypothesis be admitted, as fairly deducible from the preceding observations (17 et seq.), we must consider (for example) the secretion of the larynx and trachea, when in a state of health, as chemically differing from that poured out under the irritation of croupy inflammation, only in the different proportions of saline ingredients present in each ; and, consequently, we are not compelled to assume, in explanation of the difference of secretion, that in croup a mucous surface assumes the functions of a serous surface (*quoad* secretion).

But it may be objected to those deductions which depend upon the supposed synthesis (17) of mucus, that, according to the experiments of Dr. Babington and myself, it must be assumed that pus is *first* formed, and then carried to the secreting surface, as a pabulum for the formation of mucus ; thus making the latter a secondary product. This objection, however, can scarcely be considered as tenable ; for pus has only been used, in our experiments, for the synthesis of mucus, because it presents us with particles of albumen in a state of far finer division than can be procured by artificial means. It is, moreover, sufficiently obvious, that, in the *animal economy*, pus is not really converted into mucus ; for the former contains a large quantity of iron, which metal is nearly or altogether wanting in mucus. Is there (I would with great diffidence ask) any physiological difficulty in supposing that on the surface of a serous membrane the blood gives up a mere aqueous solution of albumen with its accompanying saline matter (serum) ; whereas on a mucous surface it parts with a mixture of its colourless albuminous particles (which have been long known to exist in blood) with serum ; whilst at the instant of their separation, or, to use chemical language, whilst in a nascent state, both combine, with an excess of saline matter ; thus constituting, according to the observations recorded in this paper, mucus, which becomes poured out on the secreting surface? On a suppurating surface, on the contrary, may we not also suppose that the blood parts with all its ingredients, excepting its colouring matter, and that portion of dissolved albumen which possesses the property of spontaneous coagulation; thus forming pus?—These views, even if their correctness be denied in a physiological point of view, are nevertheless strictly in accordance with the chemical properties and composition of blood, serum, pus, and mucus. These remarks, however, I hazard with extreme diffidence; rather wishing to place before the scientific world an account of this experimental inquiry, than to present any crude and imperfect deductions of my own; trusting, also, that the observations recorded in this paper will attract the notice of those more fitted to the task of investigating their physiological bearings than myself."

### II. SIR A. COOPER ON VARICOCELE OF THE SPERMATIC CORD.*

The indefatigable Sir Astley has communicated a good paper on varicocele in the last number of the Reports. He gives a full account of the complaint, and examines in detail its anatomy, symptoms, effects, and treatment. It is not perhaps necessary to follow the worthy and able Baronet through all his observations, which, however, we recommend to all our young, and to many of our old friends. We may cite his enumeration of the best marks by which we may discriminate varicocele from hernia.

The patient is desired to place himself in the recumbent posture: then the surgeon presses upon the spermatic cord, and raises the testis and swelling, and it disappears: he then places his fingers at the external abdominal ring, and directs the patient to rise; and if the swelling be varicose, it immediately reappears; but if it have been hernia, it cannot re-appear. Even pressure at the abdominal ring, without the patient returning to the erect position, will reproduce the swelling of the spermatic veins, by preventing the free return of the blood; but the pressure must not be sufficient to arrest the blood in the spermatic artery, or the veins will remain empty.

Sir Astley alludes to the ordinary remedies—suspension—cold bathing and evaporating lotions—the exciting of inflammation and thickening of the scrotum by blisters and means of that description—the drawing the scrotum through a ring and fixing it there—remedies useful in most cases, absolutely effectual in few or none. In some cases there is so much uneasiness and mental distress, that even amputation is demanded by the patient. Sir Astley relates a case of this description. Sir Astley speaks of ligature of the spermatic veins, but he speaks of it only to condemn it.

" But, in my Work on the Testis, published in the year 1830, I have advised the removal of a portion of the scrotum, in the following words:—

' *The removal of a portion of the scrotum will lead to a diminution of the veins of the spermatic cord; and it is an operation, in an extreme enlargement accompanied with pain, which might be tried with perfect safety, and is very likely to succeed.*'

I had, at that time, never performed the operation, and I therefore spoke of the probability of success only: but, aware of its being free from danger, and seeing that it would render the remaining portion of the scrotum a natural bandage, and that a great degree of relaxation of the scrotum also attended this complaint, and that such relaxed portion might be safely and effectually removed, I determined to take some opportunity of performing the operation.

Beside the advantage of making the scrotum, in its lessened state, a means of

---

* Guy's Hospital Reports, No. VI.

support, it must naturally occur, that the adhesion, excited by the operation, of the fascia which covers the cremaster, to the surrounding parts would produce a permanent support, and render a suspensory bandage unnecessary. It might be thought a painful operation; but it is not so, nor does it excite constitutional irritation.

The mode of performing it is as follows:—The patient being placed in the recumbent posture, the relaxed scrotum is drawn between the fingers; the testis is to be raised to the external ring by an assistant; and then the portion of the scrotum is removed by the knife or knife-scissars;—but I prefer the former. Any artery of the scrotum which bleeds is to be tied; and a suture is then made, to bring the edges of the diminished scrotum together. The patient should be kept for a few hours in the recumbent posture, to prevent any tendency to bleeding; and then a suspensory bag is to be applied, to press the testis upwards, and to glue the scrotum to the surface.

The only difficulty, in the operation of removing the scrotum by excision, is in ascertaining the proper quantity to be removed; but it adds but little to the pain if a second portion be taken away, if the first does not make sufficient pressure on the spermatic cord. It is of no use to remove a small portion of the scrotum, for from doing this I have failed. When the wound has healed, the varicocele is lessened, but not always entirely removed; but the pain and distressing sensations cease, if sufficient of the scrotum be removed.

In making the suture in the scrotum, its lower part is to be brought up towards the abdominal ring, to raise and support the testis; as does the suspensory sling when it is worn."

Sir Astley relates four cases, in which he, and one in which Mr. Key performed the operation. All the cases were attended with very beneficial results. Sir Astley observes in conclusion:—

"I wish it to be recollected, that I only recommend the removal of a portion of the scrotum in those cases of spermatocele in which the patient suffers great local pain; in cases in which he is most urgent to have the swelling and deformity of the part removed; and more especially in those instances in which the function of digestion suffers, and there is a great degree of nervousness and of mental depression. For slighter cases, a suspensory bandage must be still recommended."

### III. On a Morbid Flattening or Compression of the Left Bronchus produced by Dilatation of the Left Auricle. By J. W. King.

"A particular morbid effect," says Mr. King, "which, as far as I am informed, has not been made known, and which, as I believe, is of rather common occurrence, is the flattening and obstruction of the left bronchus, when the left auricle is dilated so as to press upon this air-tube. Our Collection affords three specimens of this affection; and I think I may say, I have remarked it many times, in different degrees."

Mr. King relates four cases, but one will be sufficiently illustrative of the fact they are intended to exhibit. We shall pick out the third case.

*Case.* Lydia P., aged 21, was under Dr. Cholmeley's treatment during two months, for a general dropsical affection, which was referred, in part, to an enlarged liver: there was evidence, however, of chest disease. She was ill altogether twenty months. She died with most parts of the body œdematous.

Some little effusion was found in the abdomen, and but a little in the thorax, with recent traces of pleuritis on both sides.

Both auricles of the heart were greatly dilated, and somewhat thickened: the left was the more capacious. The right ventricle was thick and very large, and the free edges of the tricuspid curtains thickened, and perhaps contracted. The dimensions of the left ventricle were more natural; but it was too capacious, and rather hypertrophic. All the substance of the heart was rather flabby, tough, and dark. The mitral curtains and cords were very much thickened and contracted, also firm and yellowish—certainly, the valve could scarcely close well. The lining of the left auricle was opaque, thick, and uneven. One lung was turgid, dark, and œdematous; the other was, in addition, more fleshy and lacerable, and contained several small, firm, dark, apoplexies, indistinctly bounded. Both lungs were most affected posteriorly, and their bronchial lining was much injected; the contents dark, foamy, and abundant. The left bronchus, behind the ventricle, was evidently greatly flattened. The liver was rather large, pale, coarse and fleshy, and the spleen small, firm, and darkish.

The other cases are, more or less, of the same description. In none were there distinctive respiratory signs or symptoms of the bronchial flattening, nor, indeed, would they be of much service if they existed. Mr. King concludes:—

"That the first evident degree of change is that in which the anterior surface of the tube in question forms a wide and simple plane. It can scarcely be questioned, when the tube is diminished to two-thirds, or one-half of its natural calibre—independently of permanent or transitory affections of the lining—that its defect is a very important item among the thoracic obstructions in any given case; but unless the narrowing were still more complete, a specific indication is scarcely to be looked for. I have seldom found the tube reduced to less than one-half of its natural extent; and I would merely observe, that the little advantage derivable from these notes probably depends on the illustration they afford to the general progress of diseases."

### IV. Account of a very large Calculus, passed by a Young Woman, without Operation.

Mary B. aged 18, a patient of Mr. Harris of Redruth, had suffered for seven years from symptoms resembling those of stone in the bladder. After a time, the symptoms grew more severe, and a sanguineous and purulent discharge made its appearance in the urine. Her health suffered materially.

Whilst she was sitting upon a pot-de-chambre, endeavouring by very forcible efforts to discharge her urine, and which exertions she continued for ten minutes, the calculus passed with violence into the recipient vessel.

After the stone had thus escaped, she immediately began to recover; but her health was not perfectly re-established for twelve months.

The menstrual secretion shewed itself for the first time three months after this occurrence.

*Analysis of the Calculus, by Dr. Rees.*

| | |
|---|---|
| Length .. .. | $2\frac{5}{8}$ inches. |
| Breadth .. .. | $1\frac{3}{8}$ |
| Thickness .. | $1\frac{1}{8}$ |
| Weight .. | 651 grains. |
| Nodule adherent to the calculus .. | Oxalate of lime and fusible calculus. |
| Body .. .. .. .. .. .. .. | Fusible calculus. |
| External porous layer .. .. .. | Ditto. |
| Darker lines observable in the body of the calculus .. | Lithate of ammonia. |

The nucleus consists of a porous substance, with lithates of ammonia and soda deposited within it. The porous body consists of phosphate and carbonate of lime, and animal matter. On exposing it to the action of boiling water, and afterwards to that of dilute nitric acid, a spongy matter remained undissolved, which retained the form of the nucleus. It seems highly probable that this central substance is bone.

### V. On the Effect produced upon the Pulse by Change of Posture. By William Augustus Guy, M.B. Cantab.

There is a paper on this subject compiled with great care from many and well conducted experiments. The paper is of too elaborate a description to permit us to enter into its details, and all that we can do is to present the summary given by our author, Dr. Guy, of the facts which he seems to have made out. The summary in question is short, but sufficiently explicit. Those who are anxious to become acquainted with the data upon which it has been founded, will do well to consult the original paper. That is essentially of a numerical character.

1. In healthy males of the mean age of 27 years, in a state of rest, the number of the pulse is, Standing 79, Sitting 70, and Lying 67; the difference between standing and sitting being 9 beats; between sitting and lying, 3 beats; and between standing and lying, 12 beats. When all exceptions to the general rule are excluded, the numbers are, Standing 81, Sitting 71, and Lying 66; the difference between standing and sitting being 10 beats; between sitting and lying 5 beats; and between standing and lying 15 beats. The same differences expressed fractionally, are as follow, inclusive of exceptions; $\frac{1}{9}$, $\frac{1}{20}$, $\frac{1}{6}$; exclusive of exceptions, $\frac{1}{8}$, $\frac{1}{13}$, $\frac{1}{5}$.

2. The extremes are very remote from the mean results. Thus, the greatest difference between standing and sitting is $\frac{1}{4}$, the least $\frac{1}{19}$, of the frequency standing; the greatest difference between sitting and lying is $\frac{1}{4}$; the least $\frac{1}{86}$ of the frequency sitting: whilst between standing and lying, the difference may be somewhat less than $\frac{1}{2}$, and as little as $\frac{1}{14}$ of the frequency standing. The greatest difference observed amounts to somewhat less than $\frac{1}{2}$ of the frequency standing.

3. The exceptions are as follow:—To the general law, that the pulse is less frequent sitting than standing, there is 1 exception in 12 experiments: to the general law, that the pulse is less frequent lying than sitting, there are 3 exceptions in 10 experiments: to the general law, that the pulse is less frequent lying than standing, there is 1 exception in 14 experiments. The total number of instances in which 1 or more exceptions to general rules occurs, is 34, or somewhat more than 1 in every 3.

4. The effect produced by change of posture increases as the frequency of the pulse increases.

5. The exceptions to the general rule are more numerous as the pulse is less frequent.

6. The effect produced upon the pulse by change of posture is due to muscular contraction.

7. Muscular contraction, whether employed to change the position of the body, or to maintain it in the same position, accelerates the pulse; and the effects produced by change of posture form but a particular case of this more general law.

### VI. On Hæmorrhage from the Unimpregnated Uterus, associated with Tumors of Varying Degrees of Induration and Malignancy. By S. Ashwell, M.D.

Dr. Ashwell, a zealous cultivator of his own department of science, details five cases in the present Paper, for the purpose of proving what he deems a rather novel fact; viz, that hard or fibrous tumors of the uterus, by many pathologists not regarded as malignant, may occasionally give rise to frequent, excessive, and fatal hæmorrhage. Dr. Ashwell subjoins the following points as important, in reference to these uterine tumors:—

1st—They commence in the parenchyma of the organ, or, in other words, in the substance included between its peritoneal and mucous coverings, in closer proximity to the mucous than to the peritoneal coat. Hard or fibrous tumors are more commonly deeply imbedded in the substance of the organ, and in closer proximity with its peritoneal covering; which, when they grow externally, their most common mode of increase, continues to invest them, however considerable their bulk. Hence they so rarely bleed.

2dly—When these tumors produce the hæmorrhage now described, they grow internally; not imbedding themselves in the walls of the uterus, and advancing towards its external or peritoneal coat, but, by their increase in size, distending the uterine cavity; and not only stretching and irritating the mucous membrane (thereby altering its condition and deranging its functions, and especially augmenting the quantity of the catamenial secretion), but also giving rise to morbid growth of this tissue.

"3dly—It is observed, when these tumors can be touched during life, that they are sensitive and painful, unlike polypi: and after death they are found to possess, either a laminated structure, with the hardness and the white lines indicative of a fibrous tumor; or, very occasionally being removed altogether from morbid growths of this class, they may have the stony hardness of real scirrhus, or the heterologue structure indicative of decided and undeniable malignancy. It is rare to find these hard and fibrous tumors thus encroaching on the uterine cavity. In Guy's Museum there are many preparations illustrative of organic uterine disease. In thirty examples of hard fibrous tumors, there are only three at the most four, where the growth is so placed; while there are twenty-six examples of such tumors imbedded in the various parts of the sides or walls of the organ. Thus it may, I think, be assumed, from the data now adduced, as well as from the general descriptions of these tumors, that the location of them now mentioned is exceedingly unusual; and, if I am right in my subsequent opinions, hæmorrhage from such growths will, of necessity, also be unusual. Cruveilhier and Dupuytren have probably been misunderstood by those who suppose that they regarded such an event as frequent. Certainly, if this be their conviction, it is opposed to the opinions of most pathologists."

It is to be regretted that the preceding observations are anything but lucid. The antecedent is so often shifted, that it is difficult to father it upon the relative, and it is hard to say exactly what Dr. Ashwell means to say, or wishes us to understand. We confess that we are at a loss.

The succeeding remarks are sufficiently clear, and of much practical consequence.

"Discharges of blood therefore from the uterus, continuing longer than the common losses connected with catamenial derangements, may arise, not alone from an inflammatory or congested condition of the viscus itself, independently of organic change of more than ordinary severity and protraction, from polypi, or from growths more decidedly vascular and malignant, but from hard or fibrous tumors. It is well known how rarely these growths ulcerate, excepting when they occupy the mouth or neck of the uterus; and it is still more uncommon for them, when imbedded in any part of the walls, to bleed: although I am certainly aware, that in any situation, the processes of unhealthy softening, or degeneration, and purulent secretion, with hæmorrhage, may occur. But it is a fact recently established, that these more or less hard, and occasionally malignant growths, so different in many particulars from the genuine uterine polypi, may, by having commenced, in the first instance, just behind the mucous membrane, induce morbid distention and growth of this tissue, and, by congestion and inflammation, give rise to bleedings of a continued, alarming, and fatal character: and further, after death, that this same mucous membrane may be discovered entirely free from ulceration, or even abrasion: thus tending to confirm an opinion—which, I confess I entertain—that the bleeding is principally, if not entirely furnished by the tissue covering the surface of the tumor, rather than by the tumor itself.

The peculiarity of these examples consists in the occurrence of the bleeding prior to ulceration. So that we must always bear in mind, in hæmorrhage from the unimpregnated uterus of unusual frequency, resisting the most judicious and persevering treatment, that there may be a tumor of the kind now described, distending the cavity, and out of the reach of the finger, maintaining so congested and inflammatory a condition of the mucous membrane, as almost to render these bleedings necessary for its partial relief."

Dr. Ashwell goes on to observe that a great affinity exists between hard and fibrous tumors and uterine polypi, nay, that the former are said to be not unfrequently converted into the latter, simply by descent, and the consequent formation of a stalk. Dr. Ashwell does not deny the affinity between the diseases, but he scarcely can credit the latter position. No specimen of such a change exists in Guy's Hospital Museum. That one of these hard fibrous tumors, he goes on to say, may very rarely find its way into the uterine cavity, is allowed, because the statistics of the disease prove it; and that prior to the patient's life or her powers being destroyed by the bleedings which, in this situation, the tumor may occasion, the growth may, as a *most unusual* occurrence, descend to the lowest part of the uterine cavity, distend and pass through its cervix, and ultimately find its way into the vagina, may also be conceded; but it will be a hard or fibrous tumor still; although its altered situation, and the bleedings attendant upon it, may justify and even demand its removal by the same means as in polypus.

Induration of texture is usually much more distinct in the hard fibrous tumor of the cavity than in the polypus, while the white membranous lines are much more defined and striking in it.

Many hard tumors commonly exist in the same uterus. It is rare to find more than one polypus.

In the method of growth there is a conspicuous distinction. The polypus, probably because it is not malignant, does not affect the organization of surrounding parts; the muscular walls of the uterus being rarely thickened, however large may be the polypus. The hard tumor, on the contrary, may, and often does, convert, by degrees, the uterus in its vicinity into its own diseased structure.

The internal tissue of many polypi is cellular and spongy, and copiously permeable by blood. This is not the case with the hard or fibrous tumor.

"I was much struck, a few days ago, by a preparation in Bartholomew's Museum; where a hard tumour, imbedded in the walls, had received no injection, although the vessels in every other part of the uterus were beautifully filled—a circumstance lending something more than probability to the opinion I have just now expressed, that the hæmorrhage, in these instances, is furnished by the membrane covering the tumour, and not by the growth itself; while in the polypus probably, with very few exceptions, the bleeding occurs from the vessels in its structure; as is satisfactorily proved by its texture, and by the difficulty of getting any mercury, or other injection retained in its vessels, however carefully it may be thrown in. In the polypus injected by Mr. Sibson and myself, the mercury quickly escaped through the orifices of the vessels opening on its surface.—Sir Charles Clarke affirms, that if coloured injection be thrown into the vessels of the uterus so as to make the substance of the uterus quite red, none of it passes to the tumour of fleshy or hard tubercle."

True polypus is almost invariably bereft of sensibility. The fibrous tumor is never entirely so.

Pregnancy may and often does occur in connexion with hard or fibrous tumors; rarely, if ever, when there is polypus, except where the growth arises from the cervix or os.

There is no remedy for polypus but removal. But Dr. Ashwell informs us, that—" a hard or fibrous tumour has once, in my own practice, disappeared without the use of any medicine: and Sir Charles Clarke mentions a similar case, 'where the tumour, as big as a child's head, could be felt through the parietes of the abdomen, just above the pubes: upon its surface could be felt two smaller tumours; one, the size of a man's fist; and the other twice this size.' The patient had laboured for some time under a very profuse discharge of blood from the vagina. A variety of means were employed for the relief of this case, for two years. Upon examining the abdomen at the end of this period, the tumours could not be discovered; and after death, the uterus was found as large as that of a woman at the end of the fifth month of pregnancy. Upon the anterior part of it, near the fundus, were found two small tumours, as large as peas; which were probably the same tumours before felt, of the size above mentioned; as there was no other vestige of them. These tumours were of a hard and resisting nature; and were lying between the muscular part of the uterus and the peritoneum covering it."

Such are the diagnostic marks laid down by Dr. Ashwell between the fibrous tumour and polypus of the uterus. Some are more, some less satisfactory, and it may be doubted whether any establish a true generic difference between the fibrous tumor and the former kind of polypus. But for all practical purposes they certainly are distinct maladies. The following are our author's sentiments upon their treatment.

"The ligature can scarcely be expected to produce equally satisfactory results in both diseases. The sensibility of the hard tumour, and the probability there is that a portion of the uterine structure shall be included within its grasp, will induce less favourable anticipations of decided benefit from its use. The hæmorrhage is almost invariably and permanently restrained, in polypus, by the application of the ligature; but the implication of other portions of mucous membrane than the part of it covering the hard tumour, may still maintain continued, although diminished, loss of blood.

The treatment of these cases is far from satisfactory: palliation, in most, is all which can be expected: still, the certainty in some instances, and the great probability in others, that the hæmorrhage depends on these growths will lead to more careful and protracted management. Entire abstinence from sexual intercourse—as well to avoid the certain and great danger of pregnancy, as the great yet lesser evil of excitement—must be rigidly enforced. A patient known to be thus affected, ought for years to practise such a degree of self-denial. The recumbent position, and modified but continued antiphlogistic measures, will often be demanded; and the diet, although nutritious, should never be generous and stimulating. A practitioner in these maladies will be cautious how he employs the secale, as an injection, or as an internal remedy. In my hands, it has appeared to stimulate the mucous membrane, and to increase the hæmorrhage. Narcotics, especially in the form of suppositories, have been beneficial; and the poppy and conium injections into the vagina, used cold, have appeared to restrain the bleeding. An aperient, and occasionally a purged condition of the bowels, has had a similar effect. After repeated and extensive hæmorrhage, these and other measures must be strictly pursued; nor will a disease of this nature allow the sufferer to indulge in much physical or mental exertion.

Life, in most instances, where the disease is early discovered or suspected, may be prolonged; and perhaps with a good measure of quiet and passive enjoyment, if the plan now prescribed be sedulously pursued; but on no other terms. It is possible that the tumour may temporarily cease to grow, and that the investing membrane for such period may not be the subject of repeated congestion and inflammation. Such appears to have been the result in Case, No. 1. More commonly, however, palliation and particular exemption from the bleeding is the extent of the benefit obtained.

How far iodine, aided by mild antiphlogistic treatment, may accomplish a suspension of the diseased action, I do not know; but I am favourable to its employment; nor can I think it impossible that this same agent may induce absorption."

As we observed, five cases are reported. But the generalizations on them, which we have noticed, will be sufficient for our own and our readers' purposes. Dr. Ashwell is zealous, intelligent, and practical. His observations always merit attention, and his opinions will be heard with respect.

---

### BIRMINGHAM TOWN INFIRMARY.

### Report of the Out-Patients attended by F. Ryland, Esq., between the 25th Dec. 1835, and the 25th Dec. 1836.*

The total number of cases attended in the period embraced by the Report was 1932. Of these cases 112 proved fatal. The Tables accompanying the Report, indeed, in a great measure composing it, are too voluminous for insertion here, and we shall merely pick out such remarks of Mr. Ryland's as appear to us to be interesting.

A. Mr. R. observes, that the general features of the report for 1836 resemble in a striking degree those of the reports for preceding years; the relative number of patients affected by diseases having their origin in what may be considered as external causes, such as atmospheric vicissitudes, particular occupations, or the indulgence in certain habits of life, exhibit only trifling changes from year to year; whilst in those diseases which are produced by the unascertained causes of an epidemic, each succeeding year presents to our notice great changes in the relative numbers of those attacked, and of those who become their victims.

B. *Rubeola.*—In 1835, one case of measles only was recorded, and that terminated favourably. In the present report there appear a hundred and six cases, of which nineteen, or 1 in about 5½, had a fatal termination. The causes of this mortality were various, but the most prominent was gross neglect on the part of the parents of the children attacked. The uneducated classes in Birmingham consider the eruptive fevers, especially measles and small-pox, as evils to which their children were born subject, and through which they must necessarily pass, and in consequence of this impression they seldom seek for medical assistance during the progress of these diseases till the danger is too imminent to be avoided. Another cause of the mortality of the epidemic under consideration was the simultaneous existence of hooping cough with the measles.

A third cause of the great mortality of the cases of measles was their complication with the pellicular inflammation of the fauces, pharynx, and larynx, called *Diphthérite* by M. Bretonneau. The instances in which this complication existed were numerous, and its effects were very fatal. Three children in one house were successively attacked by measles, followed by *diphthérite;* they all died within five days from the time of the appearance of the eruption.

---

* Transactions of the Provincial Medical and Surgical Association. Part II. Volume VI.

Mr. Ryland had one opportunity of examining a case of this kind after death.

*Case.* J. O. aged 5, broke out with the measles on the 8th of June. Two days afterwards Mr. R. saw him. The measles still continued out, the bowels were relaxed, the breathing accelerated and attended with a mucous rattle; the countenance was anxious, the extremities cold, and the pulse small; there was also a great difficulty of swallowing, a hoarse cough, and almost total suppression of the voice. Membranous concretions were observed on the roof of the mouth, but the back of the fauces could not be seen. The child died in the afternoon of the following day.

*Dissection, thirty-nine hours after death.*—The jugular veins were distended with fluid blood; the sub-maxillary glands were considerably enlarged. There was a thin ash-coloured membranous exudation upon the uvula, upon that part of the pharynx which is contiguous to the larynx, upon the laryngeal surface of the epiglottis, and upon the lips of the glottis as far as the margins of the ventricles of the larynx. In the last mentioned situation, the false membrane adhered rather firmly, in the other places it was but loosely connected with the subjacent parts. The mucous membrane of the epiglottis was thickened and slightly injected, that of the lips of the glottis and ventricles was much reddened. The trachea exhibited but little traces of inflammation. The lungs collapsed but imperfectly; they were in a state of sanguineous congestion, but not inflamed. The false membrane had not passed down the œsophagus, nor did the bowels exhibit any marks of disease.

Many cases of *diphthérite* in its early stages, when the albuminous exudations were confined to the tonsils, the uvula, and the palate, were speedily relieved by the topical application of a strong solution of alum, and the internal use of calomel and antimonial powder; but those cases in which the disease had reached the larynx previous to the calling in of medical aid, were universally fatal.

C. *Scarlatina.* In the two last quarters of the year 1835, there were ninety-four cases of this disease; in the first quarter of 1836, there were twenty-five cases; in the second quarter, ten cases; and in the third quarter, five; shewing the gradual subsidence of a widely spread and fatal epidemic. One half of the deaths from scarlatina occurred previously to the middle of January.

D. *Variola.* The cases of small-pox have been six times as numerous in 1836 as they were in the preceding year; more than half of them occurred in the winter quarter. The deaths are to the cases in the proportion of one to nine.

E. *Erysipelas extending to the Larynx.* G. B. aged 52, shoemaker, was attacked with erysipelas of the face, on the 10th of August. On the 14th, when the inflammation in the face had slightly diminished, the patient complained of dryness and heat about the fauces, and difficulty of swallowing; no pain or pressure in the laryngeal region; pulse frequent and weak. On the 16th, the external erysipelas had extended along the front and sides of the neck; there was great pain and difficulty in swallowing; voice weak and hoarse; occasional short cough, producing pain in the laryngeal region. At mid-day of the 17th, the patient was evidently sinking fast; the respiration was croupal, the voice reduced to a scarcely audible whisper; he could only swallow a teaspoonful of anything at a time, and a short hacking cough followed every attempt of the kind. He became comatose, soon after Mr. Ryland saw him, and died at midnight.

*Dissection, fifteen hours after death.*—The mucous membrane of the pharynx was inflamed, and upon it were two patches of coagulated lymph, beneath which the mucous membrane was much injected, and of a dark red colour. The œsophagus was healthy. The epiglottis was very much thickened, and its edges were curved backwards towards the cavity of the larynx; the membrane investing its anterior face was very tumid, of a bright red colour, and the sub-mucous tissue was infiltrated with serum. The membrane lining the posterior or laryngeal face of the epiglottis, and the whole of the mucous membrane of the larynx above the superior ligaments of the glottis, were covered with a layer of lymph, which, on being scraped off, shewed the surface beneath of an uniform dark red or nearly purple colour. The aryteno-epiglottic ligaments were thickened. The remainder of the mucous membrane of the larynx and that of the trachea were injected and covered with mucus, but the acute inflammation had not extended below the superior vocal ligaments.

F. *Strangulated Hernia.* Rather a curious case is detailed by Mr. Ryland.

*Case.* Mrs. E. æt. 86, a stout old widow, of active habits and strong constitution, had been the subject of femoral hernia on the right side for about twelve months, but had never worn a truss, and sometimes the swelling in the groin had gone up, though it was generally more or less down. It had, however, never given her any uneasiness till the 13th of March, when it suddenly increased in size, soon after which vomiting began, and the bowels became painful and constipated. Mr. R. was not sent for till the 17th; the hernial tumour was then as large as an orange, ovoid in shape, tense, rather sore, and quite irreducible. The bowels were not distended; they had not been opened since the 12th, except very slightly on the morning of the 14th: there was tenderness in the right iliac region. An enema containing castor oil was immediately administered, which brought away a large quantity of fæces, and purgatives were given by the mouth, but they were returned almost instantly; every thing that was swallowed induced vomiting. The operation was proposed and strenuously urged, but the patient and her friends would not listen to the suggestion. For the two or three following days the glysters were continued, and cold applications were used to the tumor, at the same time that gentle attempts were made to reduce the hernia. These measures were quite unsuccessful, and the patient remained unable to retain any thing upon the stomach except cold water, and gin and water, which were kept down for a few hours and then returned. A large quantity of fæcal matter was vomited. She continued in this state for many days, getting gradually weaker, but suffering no pain from the hernia, and only a little occasional uneasiness in the bowels.

On the 6th of April, the twenty-fourth day of the strangulation, she complained of hunger, and said she was being famished to death; to prevent which melancholy catastrophe she ate heartily of bread and cheese, and drank some beer, all of which she relished exceedingly. At four o'clock on the following morning, (the 7th) she passed a large quantity of flatus, and speedily afterwards a copius fæcal evacuation, of healthy appearance and of moderate consistence; and in the course of the same day she had three other motions, not quite so large as the first, but amounting altogether to a chamber-pot full. After this she became exceedingly low, and refusing all medicine, asked for gin and water. She gradually recovered completely. The hernial swelling diminished greatly in size and felt hard and rather lobulated, as if it were composed entirely of omentum.

G. In referring to the tables he has published, Mr. Ryland directs attention to the extreme fatality of infantile diseases. Of the four hundred and sixty-four patients under five years of age, fifty-eight died, giving a proportion of one death to every eight cases at that period of life. Of the remaining one thousand four hundred and sixty-eight patients, fifty-four only died, giving a proportion of one death in every two hundred and seventy-five cases in persons above the age of five years.

Of the fifty-eight deaths in children under the age of five years, twenty-nine or one half were caused by the *exanthematous* diseases; fifteen by affections of the lungs and air-passages, four by convulsions, three by fevers, two by dysentery, and the remaining five by chronic disease and accident.

Of the fifty-four deaths in persons above the age of five years, only five were occasioned by the exanthematous diseases, whilst twenty-seven or one half of the whole were caused by diseases of organs contained within the chest.

II. Mr. Ryland attempts a generalization upon some tables of the occupations of the patients. That generalization is an imperfect one, but it is an attempt. He divides the occupations into those necessarily attended by frequent exposure to the weather; those attended by continual exposure to the heat of a fire; those in which the work is performed in covered shops, and those of a sedentary nature.

| | | Bronchitis. | Chr. bronchitis. | Constipation. | Dyspepsia. | Phthisis. | Rheumatism. | Synochus. | Total. |
|---|---|---|---|---|---|---|---|---|---|
| Out-door occupations | Males | 6 | 3 | 3 | 0 | 1 | 14 | 8 | 35 |
| | Females | 6 | 6 | 2 | 9 | 0 | 11 | 3 | 37 |
| Occupations exposed to heat, Males | | 7 | 9 | 2 | 6 | 0 | 13 | 3 | 40 |
| Occupations carried on in covered shops | Males | 25 | 27 | 4 | 13 | 12 | 26 | 21 | 128 |
| | Females | 25 | 11 | 17 | 59 | 2 | 15 | 31 | 160 |
| Sedentary occupations | Males | 0 | 4 | 0 | 3 | 4 | 5 | 1 | 17 |
| | Females | 9 | 7 | 5 | 25 | 0 | 11 | 9 | 66 |
| Total | | 78 | 67 | 33 | 115 | 19 | 95 | 76 | 483 |

On the whole, Mr. Ryland deserves great credit for the pains he has taken with this and with preceding reports.

### BIRMINGHAM DISPENSARY.

A REPORT OF CASES TREATED FROM JANUARY 1st, 1837, TO JANUARY 1st, 1838. By T. OGIER WARD, M.D. Physician to the Birmingham Dispensary.

IT appears that, during the year 453 cases were treated. Of the patients, 156 were males, and 297 were females. Of the males 18 died—of the females 13. Dr. Ward must be favourably known to many of our readers, as a zealous and intelligent physician. We have not space for his entire Report, and we shall merely introduce one or two interesting facts from it.

A. *Treatment of Amenorrhœa.* " In addition to the symptoms of dyspepsia, palpitations, &c., almost every case of amenorrhœa, whether depending upon a plethoric or anæmic state of the system, was attended with more or less headache. In the treatment of this most distressing symptom, I have been guided less by a regard to the constitution of the patients, than by attention to the effects of the recumbent posture in alleviating or aggravating the pain; such patients as are relieved by lying down being almost invariably benefitted by antispasmodics and tonics, particularly iron; while those who sleep with the head raised and whose eyes are swelled in the morning, require depletion local and general."

B. *Occurrence of Fever from Putrid Exhalations.* In a preceding report Dr. Ward had stated that the number of fever patients for fifteen months was thirty-two. But in the present report, from a different district of the town, there are three deaths out of a similar number of cases, the character of which, however, was much more severe, two-thirds of them being continued fever. The river Rea, that separates Birmingham from its suburb Badesley, and serves as a "cloaca maxima" to both, carries its filthy stream onward, partly to turn a mill, and partly to fill a mill-pond, while the surplus is carried away over some flood-gates by a black stream parallel to a street which it separates from the mill-pond. During the drought that prevailed last year, the water was very low in the main stream and mill-pond, and the mills not being regularly worked, it became quite stagnant and offensive: the back stream also became dry, and shewed its mud banks, that were only occasionally wetted by a flush of the washings of the town after a shower, or by the small surplus accumulated during the cessation of the mills. The exhalations from the half dried mud and putrid water were so disagreeable at night, as to nauseate the more delicate inhabitants of the adjoining streets, and soon produced disease in the form of typhoid fever of an infectious character. Dr. Ward thinks there must have been not far short of fifty cases in all. He had seventeen under his own care, thus distributed; in one yard that looked upon the back stream, there were three slight cases in one house, all children; two severe cases in another house, a father and son, the latter of whom died; in the front house at the top of the yard were three children severely, the mother slightly, and a young man fatally affected; another mild case occurred in a child on the opposite side of the street; and a more severe one in another child at a distance along the river side; lastly, there were six cases, five of them most severe and one fatal, all in children, and one mild case in a girl, all in two adjoining houses on the bank of the river. Still lower down the stream, where the water was as black as ink, there were thirteen pauper cases in one yard, and many others, both pauper and private, along the same line. At the other side of the town, round the Soho works, where the same causes existed, fever broke out about the same time, and has continued in a very fatal form.

Dr. Ward remarks that, in Birmingham, every family, with very few exceptions, occupies a separate house. There is a difference of nearly two hundred feet in the elevation of different parts of the town, the highest points being almost five hundred feet above the mean level of the sea; and the surface is so undulating, that the drainage is excellent in every part. The streets and courts or yards in which the mechanics live are wide and airy in general; fuel is cheaper than in any other large town in England; the water used for drinking and for culinary purposes is excellent, and till within the last year there has been but little distress. Except the pearl and lead works, there are very few injurious occupations extensively carried on in Birmingham.

The type of fever was that of typhus mitior of Cullen, or the *affection typhoide* of Louis and Chomel, the most urgent symptoms being cough and diarrhœa, particularly the former. Of the seventeen cases above mentioned, eleven were below the age of twelve. The three fatal ones were a young man aged 23, a boy aged 11, and a child 1½, all of whom exhibited exactly the same lesions after death, viz., extreme congestion of the lungs, with here and there portions of red hepatization, the whole of the affected parts being softened; the bronchi red, thickened, and filled with thick mucus; the mucous membrane of the stomach injected, thickened, and softened; the same appearances in the duodenum of the two elder, with great development of the mucous follicles; the glands of Peyer prominent, and covered with sloughing ulcers, particularly at the cæcal valve; the mesenteric glands opposite the ulcerations much enlarged; the glands of Brunner enlarged and ulcerated; and the rest of the viscera healthy, but congested, except the spleen, which was softened also.

c. *Death after five weeks from perforation of the intestines.* A lad, aged 15, felt unwell from the 17th of August to the 24th, when he applied for relief. He took an aperient powder on that day, and on the 26th. Soon afterwards he complained of a fixed violent pain in the right side of his belly, which rapidly extended, while that part of his abdomen became exquisitely tender. His symptoms continued with variations and intermissions, till Oct. 3, when he died. The symptoms were pain and tenderness of the abdomen, particularly on the right side, aggravated by occasional spasms, fever, frequent vomiting, constipation, tympanitis, and rapid emaciation. He was not confined to his bed, but usually sat doubled up on a chair, as that position gave him most ease; and he was able to go down stairs the day before his death.

On dissection, there was fæcal matter in the peritoneal cavity, and there were the usual marks of peritoneal inflammation. Many ulcers with elevated edges everted externally, and penetrating its cavity, were seen on the colon. The stomach and bowels were removed and laid open. The former was injected, and its mucous membrane was softened, but that of the intestines was healthy throughout, excepting in places opposite to the external ulcers, where it was softened and perforated, forming a thin floating edge or fringe to the ulcers, but with the exception of one instance, where the perforation was two inches long by one inch and a half wide, it was as tough as natural round the ulcers; here however, it was injected and pulpy. There were four perforations in the right colon, varying in size from the largest just mentioned to half an inch in diameter, all having thickened or everted edges towards the peritoneal surface; and, besides these, there were several others laid open by the scissors in running them along the bowels. The solitary glands of the colon were enlarged, but not ulcerated. Peyer's glands were very distinct, and were spotted with black dots, but were not ulcerated in any part. The mucous glands of the vermiform process were much enlarged; and the process itself was almost divided in two parts by ulceration at about an inch and a half from its extremity. It was separated with difficulty from the matted mass of intestines.

Dr. Ward observes, and we agree with him, that there can be little doubt of the perforation of the intestine having occurred at the time of the first attack of abdominal pain.

---

### BIRMINGHAM EYE INFIRMARY.

A Report of the Cases attended during the Year 1837. By R. Middlemore, Esq.

Setting aside the numerical returns, we shall quote some of the principal facts, specifically mentioned by Mr. Middlemore.

a. *Ossification of the Crystalline Capsule.*

Mary Larkin, aged 60, received a blow upon the right eye about eight years ago, which deprived the organ of sight, but did not leave behind any manifest defect of any other description. About six months since she complained of great pain in the eye, and, on examination of the part by her surgeon, Mr. Oates, of Sutton, the lens, surrounded by an ossified capsule, was found to be dislocated.

Nov. 1, 1837.—She complains of intense pain above the eye-brow, upon the cheek bone, and towards the nose. The forehead is acutely painful, and also the back of the head on the affected side.

There is a slight zonular arrangement of vessels around the cornea, which is occasionally much increased, and it is manifest that she has suffered for some weeks past from chronic iritis. Immediately behind the cornea there is a globular body obviously covered by a white membrane, interspersed with dense yellowish-white spots; the iris is pressed backwards by the presence of this body in the anterior chamber: it was evidently the lens within the anterior chamber and surrounded by its capsule, the anterior hemisphere of which was converted into bone, but being more perfectly ossified at one part than another, the mottled and irregularly and densely dotted appearance mentioned was perceived. The removal of the ossified part was proposed, and, on the 18th, was performed, by a section of the lower part of the cornea.

The lens was of an amber colour, and was not very opaque; the posterior capsule was scarcely thicker than usual, and nearly transparent, but the anterior hemisphere of the capsule was almost entirely converted into a smooth plate of bone, except near the margin of the union between the anterior and posterior hemispheres of the capsule, where it constituted a rugged ring of bone.

The patient did well, but the sight of the eye was entirely destroyed.

B. *Treatment of Staphyloma of the Cornea.*

" James Shephard, æt. 24, sustained a severe injury to the face some months ago, which produced collapse of the right, and occasioned the following condition of the left eye:—Two-thirds of the cornea at its lower part has become prominently staphylomatous; the pupil is closed, and the iris is adherent to the upper part of the staphyloma. The eye-ball is a good deal inflamed. The objects it was desirable to accomplish in this case were, first, to lessen the size of the staphyloma; second, to remove the ophthalmia; and third, to form an artificial pupil.

As one-fourth of the cornea, and a corresponding portion of the iris were healthy, it was, I repeat, desirable to make an effort to form an artificial pupil; but, of course, before this was attempted, it was necessary to cure the staphylomatous projection by some method which would not endanger the occurrence of atrophy of the eye-ball. The use of the seton was improper on account of its great liability to produce a degree of inflammation adequate to affect injuriously the corneal or irital texture; and the removal of the projecting part by ligature or the knife was improper, by reason of their direct tendency to cause collapse of the eye-ball. The repeated tapping of the part, by means of a fine iris-knife, was not open to this objection, and, although a mode of treatment not generally to be recommended for the treatment of staphyloma, was in this instance adopted, and with perfect success; so that this person's eye is now in a fit state to be operated upon for artificial pupil."

Mr. Middlemore goes on to remark?—

" In all cases of partial staphyloma of the cornea where it is desired to leave the eye in a condition to permit the formation of an artificial pupil, in all instances where it is specially important to avoid the displacement of the lens, and the risk of producing atrophy of the globe, the operation of tapping is to be preferred, but, on account of its tediousness, and its frequent inadequacy, it is not adapted to the cure of the large and extensive variety of staphyloma, or, indeed, of any form of staphyloma the walls of which are much thickened. These last varieties of staphyloma are best treated by the removal of a small portion of the most prominent and attenuated part, as formerly explained; but, instead of using Beer's extraction knife, I now prefer to employ one the blade of which resembles the ace of spades, only narrow in proportion to its length, and having a cutting edge on either side—a sort of double Beer's knife, one edge of which, when the point is introduced within the cornea, is opposed to its upper, and the other to its lower margin."

C. *Fistulous Opening communicating with the Anterior Chamber.*

Mary E. received a blow from a cork, which was forcibly projected against the eye. In a few days afterwards, there was a small, nearly transparent, tumour just without the margin of the cornea, which contained a small quantity of aqueous fluid. On its removal, by a minute opening made with the point of a fine needle, it soon reappeared, and the iris appeared somewhat narrower on the side of this little vesicular enlargement. No astringents caused its contraction, and, when opened with a small needle, it soon filled again. Subsequently, Mr. M. applied the nitrate of silver to the part, the small swelling gradually diminished, and has not since reappeared.

This description of tumor, observes Mr. M., sometimes occurs after a small but penetrating wound at the corneo-sclerotic junction, and may either be produced by the protrusion of the membrane of the aqueous humour, or, as in this instance, by the passage of a minute portion of the aqueous humour beneath the conjunctiva. The application of the nitrate of silver to the part, after the evacuation of its contents, is usually adequate to its cure.

D. *Dislocation of the Lens through a Rent in the Sclerotica, beneath the Conjunctiva.*

W. M., æt. 21, received an injury of the right eye from a cow's horn. Four months afterwards, the eye was in the following condition:—The cornea was slightly nebulous, and somewhat conical; the anterior chamber enormously large (almost amounting to dropsy of that part); the iris was wanting at its superior part, so that the pupil resembled that of an eye in which the upper section has been made for the extraction of cataract, the surgeon having shaved off a portion of the iris in making the incision; and there was a blue mark just behind the corneo-sclerotic junction, shewing that the sclerotica, perhaps, also, the conjunctiva, had been ruptured at that part. The man could see moderately well without spectacles, but his sight was not much improved by the use of double convex glasses.

"The form of dislocation of the lens from which Manton suffered, is by no means one of frequent occurrence. It has fallen to my lot to see two instances in which the lens has passed through a rent in the sclerotica, and lodged near the corneal margin and beneath the conjunctiva. These cases came under my observation when only a very small tumour was visible, which I supposed, in both instances, to be the remains of the displaced crystalline. Although the patients suffered extreme pain for some time after the accident took place, they eventually recovered a very useful degree of vision, notwithstanding the omission, on the part of their surgeon, to remove the lens by a division of the conjunctiva. Sometimes the lens will not only pass through the sclerotica, but through the conjunctiva also, by the laceration of both membranes at the same time, and yet the patient's vision will be moderately good when assisted by the ordinary cataract glasses; but this, of course, is neither a common accident, nor the customary termination of it when it does take place.

To conclude my remarks on this interesting case:—whenever a convex tumour of considerable size and covered by the unbroken conjunctiva, forms near the corneo-sclerotic junction soon after an injury to the eye-ball, it is almost sure to consist of either effused serum or blood, or to be the displaced lens. The means of determining to which of these causes the enlargement is owing are, for the most part, abundant and manifest, and if the swelling be ascertained to arise from the presence of the lens, the rule for the guidance of our practice is absolute; the displaced crystalline must be at once removed by a division of the conjunctiva."

E. *Employment of various Remedies for the Relief of Amaurosis.*

*a. Veratrine and Aconitine Ointment.* Mr. Middlemore has tried these vaunted remedies in eight cases. His mode of using the ointment* has been to rub, daily, a quantity, the size of a small nut, above the eye-brow, by means of a bit of sponge, attached to a convenient handle, until the skin begins to smart and feel very hot.

In only *one case* was the slightest benefit obtained. It was that of a soldier suffering from dimness of vision, which, however, was not so great as to prevent him from walking with tolerable ease about the streets of the town, and managing the sale of vegetables at home. The pupil of his eye was rather large and sluggish, and there was just that sort of muddy (occasionally approaching to resplendent when viewed in a particular light) green appearance within the eye, which is noticed where chronic inflammation of the septa of the hyaloid membrane has induced a slightly turbid condition of the vitreous fluid. The pupil (and this is a very common effect of these applications) became smaller and more active, and he thinks his sight considerably improved. After discontinuing the remedy, his sight was very little better than it was before he used it.

In some cases of neuralgia of the eye-ball, Mr. M. has prescribed these ointments with unequivocal advantage.

*b. Application of the Nitrate of Silver to the Margin of the Cornea.* The use of a finely pointed stick of caustic to the margin of the cornea has been much recommended, and, some years ago, was tried by Mr. Middlemore, without advantage. His mode of applying the caustic is this :—

" Having a portion of nitrate of silver worked to a delicate point, I touch the cornea near its junction with the sclerotica so slightly as merely to produce a small eschar, on the detachment of which a minute, superficial, and perfectly healthy ulcer remains, which very readily heals and becomes imperceptible, and this I do at about four points, which are comprised within the half of the cornea."

" In one or two instances in which I have employed the nitrate of silver, a troublesome form of ophthalmia has occurred afterwards, but, inasmuch as most of the cases in which the nitrate of silver are admissible and advisable, are those in which there is an anæmic and atonic condition of the ocular tunics, and of the vascular system of the eye generally, this occurrence is very rare, and has always been, under my own observation, quite manageable."

Lately, he has had a case in which the plan has apparently been useful, and, as M. Lisfranc says that he has found it very much so, it is not altogether undeserving of attention.

*Case.* W. E., æt. 12, had suffered from an attack of fever two years previously, since which he has been blind.

The following is the state of the eyes. Pupil large, clear, and immoveable; there is no inflammation present, and his eyes are not painful. With the right eye he can just discern the degrees of light, with the other he has not the slightest perception of light. To this eye (the left) the nitrate of silver was applied four times, at suitable intervals, when the vision of the eye (formerly entirely dark) was sufficiently restored to enable him to distinguish colours, and to make out large and conspicuous objects. The pupil is smaller than it was, and the iris is more active. The use of the remedy and the improvement of vision are now progressing, though slowly.

---

* It is prepared by mixing four grains of aconitine or veratrine with half an ounce of lard.

c. *Use of Strychnia.* "I must not omit to mention that in two instances of amaurosis occurring in my private practice, I have, after the failure of other methods, placed a blister in front of the ear, and applied to the raw surface left after its removal a small quantity of strychnia, and that its use has been followed by the most satisfactory results; satisfactory, indeed, when it is considered that this method of management was tried after all the measures previously employed had failed to relieve."

### GENEVA HOSPITAL.

REPORT OF THE CASES OF THE IN-PATIENTS TREATED IN THE MEDICAL WARDS. By H. C. LOMBARD, M.D. Physician to the Civil and Military Hospital at Geneva.*

Dr. Lombard is well known in this country, as well as in Geneva. The Report from his pen contained in the last volume of the Provincial Transactions is very circumstantially, as well as ably drawn up. We regret that our narrowed limits prevent our doing more than present the brief summary of facts with which it terminates, a summary, however, which, though brief, enables us to understand at a glance the principal features of the Report itself.

" If we now," says Dr. Lombard, " collate the different facts contained in this paper, and endeavour to ascertain the frequency or the rarity of the diseases we have been considering, with reference to the climate of Geneva, and to the influence of the different seasons, we may trace the following summary of the influence of the seasons on the development of certain diseases.

1*st. Winter* (*December, January, and February*).—During this quarter, which may be considered as the coldest in the year, the number of patients admitted into the hospital is in general pretty considerable, without, however, being the fullest season of the year. The prevailing maladies during these three months are pneumonia, catarrhal fevers, acute muscular rheumatism, and apoplexy. The diseases which are met with more rarely in winter than at any other period of the year, are bilious and intermittent fevers and acute articular rheumatism; but we must remember that, in the last of these complaints, the differences in each season are not in general very remarkable.

2*nd. Spring* (*March, April, May*).—This period of the year is composed of two rainy months, and of one month (March) in which the temperature is sometimes high and sometimes cold, so that altogether the season may be considered as damp and variable. The number of entries in this season is not so considerable as in the other quarters, for it is only the acute and chronic pulmonary catarrhs which attain their maximum of frequency during the spring, whilst apoplexy, erysipelas, typhoid fevers, and acute articular rheumatism, are more unusual during the spring than at any other period of the year.

3*rd. Summer* (*June, July, and August*).—The three hottest months in the year are those which furnish the greatest number of patients, by which I do not mean that the number of patients assembled at the same time in the hospital is the most considerable at this period of the year, because in the hot season diseases are usually shorter and more acute, and convalescence less tedious; so that during the summer we have often had fewer patients at a time than during the winter, though the number of entries has been more considerable in the former than in the latter season.

The prevailing complaints in summer are, erysipelas, acute articular rheumatism, sciatica, and bilious, typhoid, and intermittent fevers. Those most rarely

* Provincial Transactions, Vol. VI. Part II.

met with in summer are, acute muscular rheumatism and chronic pulmonary catarrh.

4*th, Autumn* (*September, October, November*).—A gradual decrease of temperature, fogs, and rainy weather, characterise this period of the year. This season, which, to judge of it from our own sensations, and from the generally received opinion, would appear very unhealthy, is on the contrary the time of year in which we meet with the smallest number of patients, and the lowest mortality.

One disorder only, acute articular rheumatism, appears to have a certain degree of frequency in autumn, though its maximum does not belong to that season alone, since the same amount of frequency is observed in summer. The diseases which are met with more rarely in autumn than at any other period of the year, are acute pulmonary catarrh, pneumonia, sciatica, and catarrhal fevers."

---

### PHILADELPHIA HOSPITAL.

Clinical Report of the Surgical Department, for the Months of May, June, and July, 1837. By W. E. Horner, M.D. Surgeon to the Hospital.*

From the Report before us, we extract a few of the main particulars.

A. *Treatment of Fistula Ani by the Seton.* "The common mode of treating this disease now is by a division of the sphincter ani. My own impressions are in favour of the seton, and the cases treated were in that way. The time was too short to exhibit results; but from preceding experience, and especially when the constitution is impaired, it appears to me to be the best of the several modes."

It will take, we think, not a little persuasion, to induce surgeons to abandon their present operation and employ the seton.

B. *Treatment of Leucorrhœa, by Stuffing the Vagina with Lint.* "The results of a treatment of three cases of women of the town, whose leucorrhœas it is extremely difficult to distinguish from blennorrhagia and the reverse, excited strong hopes that this obstinate affection is susceptible of an improved mode of cure, the first idea of which originated, I believe, in the French capital; to wit: that of cleaning out the vagina well every day with some abluent, and then packing it full and systematically with lint, by the aid of a speculum; the first step of the dressing being to keep off the lips of the womb from the vagina, by filling up the connecting depression."

The detergent used in one of the cases was the liquor sub-acet. plumb., and the vagina was afterwards plugged with cotton. An ulcer existed at the beginning on a lip of the womb. This patient, from being stationary previously, improved rapidly under the treatment.

A second patient was cured very rapidly and perfectly, by the daily plugging of cotton, and cleansing with soap and water. The treatment lasted about two weeks, at the end of which time she was seized with symptoms of inflammation of the womb, which also got well. Whether the latter disease was a consequence of the treatment, or merely a concomitant of the other, cannot be determined until more cases managed in this way are brought before the profession.

A third case was cured in eighteen days, and without accident, by daily plugging with cotton and washing the vagina clean.

---

* The American Journal of the Medical Sciences. November, 1837.

c. *Treatment of Acute Gonorrhœa by Cubebs.* In this affection the symptoms being so distinct as to leave no doubt of their nature: the piper cubeba exhibited the most marked and incontestable influence. In nine cases seven were cured on an average treatment of ten or twelve days; an eighth was relieved, but not cured; and in the ninth the remedial value was unsettled, perhaps nugatory. The plan of treatment was to resort to blood-letting, when there was plethora, and the pulse full; to open the bowels freely with a saline cathartic, to keep the patient on a vegetable diet; and then to administer a drachm morning, noon, and night of the powdered cubebs, directing during the continuance of this course, to use as little water or diluent drinks as possible.

The latter recommendation certainly runs counter to the ordinary method. And yet it is by no means opposed to common sense or to received principles. One of two things is desirable—either for the patient to make very little water—or to make a good deal of a very dilute description. Which of the two is best it is not easy on theoretical grounds to determine. By the one the parts are kept quiet, but the urine, when it does pass, is very acrid—by the latter, the parts are not kept so quiet, but the urine is less stimulating. For ourselves, we confess that we have so constantly given diluents that we cannot speak practically on the subject. But of one thing we are sure, that Dr. Horner's recommendation of cubebs is overdone.

D. *Proposal for a New Treatment of Vesico-Vaginal Fistula.* Dr. Horner proposes to bring the *womb* so far down in the vagina that its anterior face may be so fixed as to supply the loss of the *inferior fundus* of the bladder and *its neck*. This idea was carried into execution in the following case.

*Case.* Catherine Hurley, æt. 30, suffered, in parturition, a slough of the greater part of the vesico-vaginal partition. Dr. Horner adopted the following process.

A silver instrument of four incees in length, resembling a female catheter, but having a broad circular shoulder in the middle, was provided for the bladder. Another instrument, founded upon the idea of the ephelcometre of Mr. Guillon, and resembling in its construction an umbrella-frame deprived of all its arms but two, and they cut off beyond the second joint, was provided for the uterus. It was made to expand like an umbrella and to close in the same way: and when expanded, it assumed at its upper part a triangular form, of the shape and dimensions of the cavity of the womb, but when closed, it was a cylinder of three lines in diameter. This instrument, when closed, could be readily introduced into the womb and afterwards expanded there, which expansion gave a complete command of the position of the womb, for by the handle of the instrument the womb could be drawn down at pleasure or shoved back, or, in fact, directed in any way.

The first instrument being introduced through the urethra into the bladder, after the second was expanded in the womb, the bladder was shoved back by the shoulder of the catheter, and the womb brought down by the ephelcometre. The handle of the latter was carried through a hole in the shoulder of the catheter, and fixed in its proper position, so as to be stationary. The relative position of the womb and bladder was now such that the womb plugged up the opening in the fundus of the bladder, and it only required the surfaces in contact to form adhesions for a cure to be the result. To facilitate those adhesions, the edges of the fistula were touched with lunar caustic.

Catharine Hurley wore this apparatus for two days without much inconvenience; there were, however, some mechanical defects in the construction of the catheter, which made it more painful than it need be, and Dr. H. took it out to amend it. But she became unmanageable and finally declined the treatment.

We have great doubts both of the efficacy and safety of this plan.

### WESTERN LYING-IN HOSPITAL AND DISPENSARY, ARRAN QUAY, DUBLIN.

Second Medical Report. By Fleetwood Churchill, M.D. &c. &c.*

This Report, like Dr. Churchill's preceding one, is characterized by great clearness, precision and ability. We shall select a few particulars for notice.

The Report embraces the period comprised between the 1st of November 1836, and the 31st of December 1837.

During that time, 391 females derived assistance from the hospital: of these, 128 were intern; and 263 extern patients.

From this number we must deduct twenty-one cases of abortion, and one case of uterine hydatids, which will leave 369 cases of labour.

The number of children born amounted to 376, of whom 206 were males, and 170 females; twenty-eight of them (seventeen males, and eleven females) were still-born, or died an hour or two after birth.

Of these 5 were premature—2 were breech presentations—2 were footling ditto—2 were funis ditto—1 was a footling case, with prolapsed funis—1 was covered with syphilitic eruption—1 was a crochet case.

In 299 cases, the duration of labour was accurately noted. In 65 cases it was under 6 hours—in 93, under 12 hours—105 under 24—18 under 36—7 under 48—5 under 60—5 under 96—1 under 120.

In almost every tedious case, no application was made for medical assistance till the patient, or her friends, were alarmed by the delay.

In 358 cases, the presentation was as follows:—In 331, it was natural—in 4 the hand descended with the head—in 9 the breech presented—in 10 the feet, in three of which the funis prolapsed—in 2 the funis presented—in 2 the arm.

There were seven cases of twins; their sexes were:—

| | | |
|---|---|---|
| In 1st case...... | 2 males. | 0 females. |
| 2nd ........ | 2 .. | 0 .. |
| 3rd ........ | 1 .. | 1 .. |
| 4th ........ | 2 .. | 0 .. |
| 5th ........ | 1 .. | 1 .. |
| 6th (premature) | 0 .. | 2 .. |
| 7th ........ | 2 .. | 0 .. |
| | Total, 10 | Total 4 |

Of these, one male and two females (premature) were still-born, or died immediately after birth.

As to the presentations; in four cases both the children presented naturally, and one child was still-born. In the fifth case, one child presented the breech, and the other the arm. In a sixth case, one child presented the breech, and the other the head: in each of these cases both children lived. In a seventh case, the presentation of one child was natural, and the hand of the other descended along with its head: both children were lost.

No cases of puerperal fever occurred during the year either among the in, or the out-patients.

Of the 391 females attended in the hospital or at their own homes, three only died (or one in 130), and two, at least, of these, owed their deaths to circumstances which could never have occurred had they been delivered in the hospital. The third sunk from the shock of a tedious labour upon a constitution enfeebled by severe pulmonary disease.

* Dub. Journ. May, 1838.

*Tedious Labour.* "In the Report of the Hospital for 1835-6, I took occasion to allude to the question of how far the length of the first stage of labour may influence the duration of the second stage, and the consequences to the mother and child. Taking the rupture of the membranes as the limit of the first stage, (which is generally, though not always, the case,) and referring to the tabular views I gave of the intervals from the commencement of labour to the rupture of the membranes, and from the rupture of the membranes to the birth of the child; I drew the following conclusions:

1st. That the length of the period after the evacuation of the liquor amnii, bore no proportion to the time which elapsed previously. 2nd. That the constitutional effects of labour are not to be estimated by the length of the first stage, &c."

Dr. Churchill subjoins four tables—the first comprising nine cases of labour of thirty-six hours duration—the second, four cases of forty-eight hours duration—the third, six cases of sixty hours duration—the fourth, three cases of ninety hours duration. He adds:—

"This table appears quite conclusive as to my first proposition; for out of twenty-one cases of labour, varying in duration from thirty-six to ninety-six hours, in only four, did the second stage amount to more than four hours, while in eleven it was concluded in one hour. Neither did the duration of the second stage increase in proportion to the prolongation of the whole labour, for of the three cases of ninety-six hours each, in only one, did the second stage exceed three hours.

These cases also afford at least negative evidence in support of the *second* proposition, for although in all but one, it was the prolongation of the first stage which caused the delay, and although in some this first stage was both excessively long and badly managed, (from being intrusted to ignorant females,) yet in no instance did the constitutional symptoms which characterize powerless labour arise; and this I fully believe, because it was during the first, and not the second stage, that the delay took place."

"So far as the series of facts I have just presented extend, they are in direct opposition to the recorded opinions of Professor Hamilton; for a prolonged first stage neither rendered 'the powers of the uterus inadequate to expel the infant with safety to its life, or to the future well-being of the patient;' 'nor disposed the uterus to contract irregularly, so as to occasion retention of the placenta;' 'nor too feebly to prevent fatal hemorrhage;' 'nor lastly, did it give rise to febrile or inflammatory affections of a most dangerous nature.'

For 1st, all the children were expelled alive and continued to live, except two, one of which was premature (six months), and the other presented with the funis, and whose deaths were consequently not attributable to the protraction of labour. 2ndly, neither flooding, retention of placenta, fever, nor inflammation, happened in any case; on the contrary, every one of the cases recovered as well as after an ordinary labour of twelve hours' duration."

As for poor Professor Hamilton he must now be accustomed to seeing facts versus his dogmatic assertions. But he has run a-muck at every body so often, that he is looked on by all as an Accoucheur-Quixote.

---

### NEWRY DISPENSARY AND FEVER HOSPITAL.

REPORT FOR THE YEAR, 1837. By J. MORRISON, M.D. PHYSICIAN TO THE HOSPITAL.

We are very glad to perceive the disposition so general on the part of the medical officers of our Irish charitable institutions, to publish efficient Reports. There is no lack of energy and talent in the Green Isle. We regret that our crippled space this quarter, compels us to limit our notices of these Reports. We can only select an insulated fact or two, from Dr. Morrison's.

1. *Treatment of Influenza.*—It was generally found that, with the exception of aperients and diaphoretics, the usual antiphlogistic measures, even at the commencement of the disease, could not for any length of time be safely employed, their effects being soon recognizable as very different from those which accrue from their administration in pure inflammatory affections of the respiratory organs.

"After the first two or three days of the incursion, there was seldom any risk in using tonics and stimulants; and this treatment I found to be by far the most successful. Under the tonics, I would comprise nutritive broths, wine, and, in some cases, the preparations of iron, of bark, &c. And under stimulants, the decoction of polygala seneka, in conjunction with carbonate of ammonia, and camphorated tincture of opium, as well as blisters, mustard cataplasms, rubefacient liniments, &c."

In some cases, when great debility existed, with excessive secretion from the bronchi, acetate of lead seemed to form a highly valuable adjuvant to the list of internal remedies. Several persons, labouring under "suffocative catarrh," certainly appeared to be rescued from the jaws of death by the administration of an emetic of mustard, and this followed up by a combination of acetate of lead and camphorated tincture of opium, together with external stimulation over the chest.

2. *Priapism in Typhus Fever.*—"I wish to advert to a symptom which was manifested in some five or six cases, and which I am not aware of as having been previously observed in this country—I mean priapism. My attention was directed to it, in the first two cases, by accident; but since these, I have inquired for it, and found it to exist, under certain circumstances, rather frequently. In two of the cases in which it existed, there was considerable heat at the lower and back part of the head. Leeching to the occiput, followed by cold lotions and blistering, together with calomel and emetic tartar, and morphine draughts at bed-time, in these removed the affection. In the three or four other cases, no unusual heat at the back of the head was felt; and in these, blistering, succeeded by tincture of opium and emetic tartar, also completely removed the priapism, though one case terminated fatally; and unfortunately no examination would be permitted."

3. *Successful Amputation during Spreading Gangrene.*—"A young man received a gun-shot wound in the ham. On visiting him a few hours afterwards, I found the foot and leg cold, and without any appreciable pulsation; no hæmorrhage of consequence had taken place. Believing the popliteal artery to be wounded, and amputation of the limb necessary, I immediately sent for further advice. A medical friend soon arrived, but whose opinion of the case did not coincide with mine. He urged the propriety of attempting to save the limb. Mortification manifested itself on the foot in a few days, and very soon after became master of the leg. The patient's strength was now rapidly failing, his pulse 130, and like a thread, his countenance almost cadaverous, and the *disease quickly extending upwards.* As a *derneir* resort, amputation was now decided on at a numerous consultation. I accordingly removed the limb about six inches above the knee, though in the full expectation of finding the man dead the following morning. The next day, however, it was found he had passed a good night, felt and looked better, and from this date he gradually recovered."

On examining the limb, the popliteal artery was found completely lacerated, the soft parts below the knee were sphacelated, and those above it, almost as high as the place of incision, injected with a greenish serum.

We quite agree with Dr. Morrison that a stronger proof than this case could not possibly be given, of the occasional propriety of amputation in spreading gangrene.

4. *Cure of Wounded Brachial Artery by Pressure.*—A man received the stab of a sharp-pointed instrument, which wounded the brachial artery, about a hand's breadth above the elbow-joint. The blood gushed out *per saltum*, and a large quantity was lost in a very short time. The wound being small, Dr. M. resolved to try pressure, having formerly treated a similar case with success. A graduated compress was accordingly applied over the wound, and the limb from this to the fingers pretty firmly enveloped by a bandage. A short distance above the wound, another compress was adjusted over the brachial artery, for the purpose of impeding the current, but was made to project considerably above the surface of the integuments, so as that the bandage which retained it would admit of a free collateral circulation. The man was kept in bed, lived on slops, and took digitalis for eight or ten days, when the wound was perfectly healed, and the artery apparently in its natural state.

